# From transformation to chronification of migraine: pathophysiological and clinical aspects

**DOI:** 10.1186/s10194-020-01111-8

**Published:** 2020-04-29

**Authors:** M. Torres-Ferrús, F. Ursitti, A. Alpuente-Ruiz, F. Brunello, D. Chiappino, T. de Vries, S. Di Marco, S. Ferlisi, L. Guerritore, N. Gonzalez-Garcia, A. Gonzalez-Martinez, D. Khutorov, M. Kritsilis, A. Kyrou, T. Makeeva, A. Minguez-Olaondo, L. Pilati, A. Serrien, O. Tsurkalenko, D. Van den Abbeele, W. S. van Hoogstraten, C. Lampl, On behalf of School of Advanced Studies of European Headache Federation (EHF-SAS)

**Affiliations:** 1 Headache and Craniofacial Pain Unit, Neurology Department, Hospital Universitari Vall d’Hebron, Headache and Neurological Pain Research Group, Vall d’Hebron Research Institute (VHIR), Departament de Medicina, Universitat Autònoma de Barcelona, Barcelona, Spain; 2 Headache Center, Child Neurology Unit, Bambino Gesu’ Children’s Hospital, Rome, Italy; 3 Juvenile Headache Centre, Department of Woman’s and Child’s Health, University Hospital of Padua, Padua, Italy; 4 Department of Internal medicine, Sant’Andrea Hospital, University of Rome, Sapienza, Italy; 5 Division of Vascular Medicine and Pharmacology, Department of Internal Medicine, Erasmus University Medical Center, Rotterdam, The Netherlands; 6 Department of Biomedicine Neuroscience and Advanced Diagnostics, Policlinico Paolo Giaccone Hospital, University of Palermo, Palermo, Italy; 7 Headache and Craniofacial Pain Unit, Neurology Department, Hospital Universitario Clínico San Carlos, Madrid, Spain; 8 Neurology Department, Hospital Universitario de La Princesa & Instituto de Investigación Sanitaria de La Princesa, Universidad Autónoma de Madrid (UAM), Madrid, Spain; 9 Department of Clinical Neurology and Sleep Medicine, The Nikiforov Russian Center of Emergency and Radiation Medicine of EMERCOM of Russia, Saint-Petersburg, Russia; 10 Grevena General Hospital, Grevena, Greece; 11 University Hospital of Psychiatry and Psychotherapy, University of Bern, Switzerland University Hospital of Psychiatry, Bern, Switzerland; 12 Headache Unit, Department of Neurology, Medical center “New Medical Technologies”, Voronezh, Russia; 13 Department of Neurology, Universitary Hospital of Donostia, San Sebastian, Spain; 14 Department of Neurology, Clínica Universidad de Navarra, Pamplona, Spain; 15 Department of Neurology, Hospital Quironsalud Donostia, San Sebastian, Spain; 16 Department of Neurology, University Hospitals Leuven, Leuven, Belgium; 17 Department of Neurology and Neurosurgery, State Institution “Dnipropetrovsk medical akademy MOH Ukraine”, Dnipro, Ukraine; 18 Department of Neurology, Ghent University Hospital, Ghent, Belgium; 19 Department of Neuroscience, Erasmus University Medical Centre, Rotterdam, The Netherlands; 20 Headache Medical Center Linz, Ordensklinikum Linz Barmherzige Schwestern, Linz, Austria

**Keywords:** Chronic migraine, Transformation, Pathophysiology, Risk factors, Genetics, Biomarker, Neurophysiology, Neuroimaging, Animal models

## Abstract

Chronic migraine is a neurological disorder characterized by 15 or more headache days per month of which at least 8 days show typical migraine features. The process that describes the development from episodic migraine into chronic migraine is commonly referred to as migraine transformation or chronification. Ample studies have attempted to identify factors associated with migraine transformation from different perspectives. Understanding CM as a pathological brain state with trigeminovascular participation where biological changes occur, we have completed a comprehensive review on the clinical, epidemiological, genetic, molecular, structural, functional, physiological and preclinical evidence available.

## Background

Migraine is a neurological disorder characterized by attacks of throbbing headache and neurological symptoms such as nausea, vomiting, hypersensitivity to environmental stimuli and mood changes. The development and course of migraine differs from patient to patient, where a subset of patients experience an increase in frequency over a period of months or years [1]. This process may lead to a chronic form of migraine that, according to the International Classification of Headache Disorders (ICHD-3) [2], is called chronic migraine (CM). This form of migraine is characterized by 15 or more headache days per month of which at least 8 days per month show typical migraine features, for at least 3 months. In 1982, Mathew et al. reported a series of patients with a clear-cut past history of distinct attacks of migraine whose headaches evolved over the years into a daily or near daily problem [3]. He was the first who proposed the term “transformed migraine”.

Migraine transformation or chronification clinically represents a more or less consistent increase in migraine frequency until, in most of the cases, it develops into a constant migraineur state with very frequent, disabling headache with associated symptoms, increased use of acute medication, high medical care and reduced quality of life. Headache interferes with life, work and results in a high burden of the disease.

The estimated prevalence of CM worldwide ranges widely between 0.9% to 5% [4]. CM prevalence is three times more common in women than men (18.9% vs. 9.8%) and presents two peaks between ages of 18–29 and 40–49 years-old [5, 6]. The development from EM to CM is estimated to occur in approximately 2.5% of the patients with EM per year, while only a limited proportion with CM revert back to EM [5, 7].

Underlying this process, central and peripheral neurological functional and even structural changes are occurring. Many studies tried to identify factors associated with migraine transformation utilizing different approaches. To establish the clinical risk factors for chronification and the structural or functional neurological changes that occur in patients who evolve to CM studies should include large migraine cohort studies with long-term follow-up. Due to the evident complexity of these studies, the majority of studies have tried to address this question with retrospective approaches or by comparing cohorts of EM and CM patients.

A better understanding of these underlying pathophysiological changes in light of the accompanying clinical developments, could possibly help us to discover new disease markers, or even future treatment targets. Subsequently, the objective of this review is to present the current knowledge on clinical and pathophysiological signatures of CM in an attempt to unify the two different perspectives.

### Clinical characteristics

The development into CM does not occur in all patients with EM [8]. Therefore, identifying risk factors associated with migraine transformation/chronification may provide crucial information in understanding the underlying mechanisms [7]. Epidemiologic studies have described clinical factors that are more common in CM patients compared to EM patients [9, 10]. Although it has been described a statistically relevant association between CM and demographic, lifestyles, comorbidities or other migraine features [5], the underlying pathophysiological mechanisms remain to be elucidated.

Clinical risk factors for migraine transformation can be divided into non-modifiable and modifiable risk factors. Non-modifiable risk factors mainly include sociodemographic features. Modifiable factors, which can provide targets for intervention, include lifestyle factors, headache features and comorbidities [7, 9].

#### Demographic factors

The most important non-modifiable risk factors for developing CM include age, sex, race, socioeconomic and educational status [11].

Women tend to have a greater risk for chronification than men, even when adjusting the data for medication use and headache frequency [12, 13]. Additionally, according to the American Migraine Prevalence and Prevention (AMPP) Study [14] and the International Burden of Migraine Study (IBMS) [15] both EM and CM are more common among women and young adults. Surprisingly, a recent study found that the typical risk factors (demographics, headache features, and comorbidities) predicted the chronification in men less accurately. This implies that prognostic factors of chronification might not be as well understood in men than in women [16]. Similar sex correlations also seem to exist in the adolescent population, since the incidence of chronic daily headache and frequent migraine is higher in girls than in boys [17, 18].

A pattern of increasing CM prevalence with age from 18 to 50 year-old has been observed for both males and females [6]. Regarding race, although both CM and EM respondents were more likely to be Caucasian, a larger proportion of CM patients was Caucasian [9, 19, 20].

Less well characterized is the relation between lower education status and CM. The majority of studies have found that patients with CM have lower levels of education compared to EM [5, 19, 21]. However, the AMPP and IBMS studies found no significant difference with regard to level of education [9, 15]. Furthermore, CM patients were less likely to be employed full time, and more likely to be occupationally disabled [9, 15, 19]. In the same study differences regarding marital status have also been reported, although the majority of both groups were married and there were not any group with a conclusive higher risk [9]. Relating to these characteristics in adolescents with CM, high prevalence of chronic daily headache (CDH), a diagnosis partially including CM patients, has been shown to be associated with lower household economic status and acute family financial distress [17]. On the other hand, the Frequent Headache Epidemiology study could not confirm any correlation between onset of CDH and age, sex, marital status, educational level, and race [22]. Although numerous studies have found that CM patients tend to have lower levels of education than EM patients, no definitive conclusion can be drawn due to replication issues.

#### Lifestyle

The identification of modifiable risk factors may provide targets for future interventions in order to avoid chronification. Among these are caffeine misuse, body weight gain, and sleep disorders [7].

It has been shown that inappropriate high caffeine consumption increase the risk of progression into CM [23]. In fact, subjects with CDH were more likely to have been high caffeine consumers before the onset of CDH [23].

Comprehensive studies have investigated the association between migraine and obesity. Some population studies show a strong positive association between obesity and headache frequency in obese women [24, 25]. Moreover, a recent meta-analysis of available observational studies suggests an increased risk of having chronic migraine in obese and pre-obese patients compared with normal weight subjects [26]. This association seems to also exist between body weight and other non-migraine headaches which questions whether there is a direct causal link between body weight and CM.

Poor sleep quality and sleep disorders are considered risk factors for migraine transformation. The Chronic Migraine Epidemiology and Outcomes (CaMEO) study showed that CM patients more frequently reported sleep apnea or were more likely to be at “high risk” for sleep apnea than EM patients [27]. CM patients showed poorer sleep quality compared to EM patients with higher rates of sleep disturbance, snoring, shortness of breath, somnolence and sleep adequacy [27]. The relationship between obstructive sleep apnea and migraine progression are not clearly understood but some physiological changes as fluctuations in intracranial and arterial pressure during snoring, hypoxia, hypercapnia, fragmentation of sleep and increased muscle activation during awakening during apnea may underlie this relationship [28].

For the evidence set out before, lifestyle most likely plays a role in migraine chronification. Consequently, dietary measures to minimize caffeine consumption and weight gain, exercise and sleep regulation strategies should be considered for prevention of migraine transformation.

#### Comorbidities

Patients with CM significantly more often reported comorbidities than patients with EM such as psychiatric disorders, head and neck injuries, cardiovascular disease, metabolic syndrome, asthma, sleep apnea and other pain syndromes [5, 7, 29]. If untreated, these comorbidities can increase the risk for migraine chronification and migraine-related disability, leading to a decrease in the quality of life and adversely affect the treatment outcomes [28, 30, 31].

In the CaMEO study furthermore it was shown that all comorbidity classes were associated with a statistically significant risk of progression to CM. However, the group of subjects with the most comorbidities were approximately 5 times more likely to progress to CM than subjects of the fewest comorbidities class [32].

Psychiatric comorbidity is particularly relevant in the group of patients with CM. CM is more common in women with severe depressive disorders [28, 31], and has been shown to be strongly associated with moderate and severe depression [29]. These associations are also highly relevant in the larger picture, as the effects of depression, anxiety and obesity are additive [5].

A variety of psychological and personality traits are also discussed as risk factors of migraine progression. Major life changes, such as divorce, marriage, or change of employment status, can exacerbate symptoms and headache frequency, increasing the risk of chronification [33]. Also, posttraumatic stress disorder [34] and certain personality profiles, particularly obsessive-compulsive, dependent, avoidant, and passive-aggressive are of prognostic significance [35].

Chronic pain disorders, including fibromyalgia, back pain, and neck pain, are more common in people with CM than EM [36]. Non-cephalic pain may be used to identify people with EM at risk of the onset of CM and people with CM at risk of persistent CM [36].

Finally, cardiovascular disorders including heart disease/angina, stroke and cardiovascular risk factors including high blood pressure and high cholesterol occurred with greater frequency in CM than EM patients [9, 20].

#### Headache features and treatment

Headache frequency is one of the most important risk factor for progression from EM into CM [8]. The risk increases with increase of headache frequency in a non-linear fashion, where a minimum of 3 headaches per month was associated with an elevated risk for new-onset of chronic headache [22]. Although the threshold for CM has been set at 15 headache days/month, a clinical study [10], showed that patients suffering from 10 or more headache days per month showed less clinical differences with CM patients than those with lower frequencies suggesting that chronification is already notable in patients with high frequency EM.

One of the most interesting headache features in CM patients is cutaneous allodynia. This reflects the perception of pain in response to non-noxious stimuli and may be considered a clinical marker for central sensitization [37]. Cutaneous allodynia affects 63% of migraineurs in the population and is associated with frequency, severity, disability, and associated symptoms of migraine [38]. In a prospective study [39] allodynia was an independent predictor for increase in number of migraine days and migraine chronification. This also has therapeutic implications. Migraine patients who describe the presence of allodynia during their attacks, should be treated within 30 min from attack onset with triptans [40].

Other well-known and established risk factors for migraine transformation is medications disuse that includes medications overuse and ineffective treatment of migraine attacks. Symptomatic medication overuse is believed to play a major role in progression from EM to CM. Acute medication overuse is defined as intake of analgesics on 10–15 days per month. It can cause rebound-drug-induced headache, therefore transforming self-limited headaches, and particularly migraine, into chronic headache [41]. The counter-proof of this concept is that withdrawal from the overused medication leads to lower headache frequency and less disability [42]. Among overused drugs, opioids and barbiturates are associated with dose-dependent increased risk of new-onset CM, while triptans induce migraine progression only in those with high frequency at baseline, but not overall. Nonsteroidal anti-inflammatory drugs (NSAIDs) protect against migraine progression unless individuals have 10 or more headache days per month [26]. The most effective way to prevent medication overuse headache is to identify patients at risk and to educate them about the use of acute medication. The risk is higher in patients with frequent headaches, use of opioids and tranquilizers and comorbid anxiety and depression [41].

On the other side, ineffective treatment, and the consequent insufficient acute pain relief, can also lead to central sensitization, which can further lower the threshold of migraine attacks and promote chronification. Inadequate acute treatment efficacy was also associated with an increased risk of new-onset CM [43]. Patients using NSAIDs and simple analgesics were less likely to be in the high treatment efficacy categories than patients who used triptans [43]. Moreover, acute treatment is less effective in patients with CM than in patients with EM, patients with more severe attacks, allodynia, comorbid depression and medication overuse headache [44]. For this reason, rapid and complete treatment of the migraine attack is a crucial intervention to prevent migraine transformation.

### Genetics and epigenetics

Genetic factors seem to be a component in determining the risk of developing EM with and without aura [45]. However, the role of a genetic influence on the progression of EM into CM remains to be elucidated [46]. The number of studies that specifically assess genetics in CM is very low and the relevance of their findings has to be interpreted with caution.

According to the scarce studies published on the possible genetic link to migraine chronification, three groups of genes have been proposed: genes potentially linked to migraine or pain progression, genes potentially linked to addiction and analgesic overuse, and other genes involved in neuronal hyperexcitability or oxidative stress [47]. Catechol-O-methyltransferase (COMT) polymorphisms could be implicated in the predisposition to chronic pain conditions [48]. Previous reports indicate that COMT polymorphisms are associated with susceptibility to EM [48], but no specific studies in CM have been conducted. A variety of potential candidate genes in drug addiction have been shown to possibly play a role in migraine chronification, especially in patients with analgesic overuse [47]. It is remarkable that some of these genes involved in serotonergic and dopaminergic pathways, also have been described to play a role in migraine pathophysiology [49, 50]. Oxidative stress is a subject increasing in popularity regarding its relation to the pathophysiology of migraine. However, a study that investigated 10 polymorphisms in 8 oxidative stress-related genes in a small population of CM patients did not detect a relationship with CM [51]. However, as migraine is considered a complex disease with multifactorial inheritance, Genome-Wide Association Study (GWAS) seems a more appropriate approach to study migraine genetic background. To date, 4 GWAS studies [52–55] and 3 meta-analyzes [56, 57] have been performed in EM patients leading to the identification of 44 single nucleotide polymorphisms (SNPs) on 38 distinct genomic loci associated with migraine, mainly involved in vascular and neural function [58]. Although the number of SNPs identified as associated with EM has steadily increased, our knowledge of CM genetics remains considerably poor. Studies on the genetic association of several SNP tests failed to provide significant genetic risk factors for the development of CM. The first comprehensive study on genetic association in CM and high-frequency migraine, tested 144 SNPs from 48 genes in 1019 patients with CM or high-frequency migraine, without finding significant associations [52]. Since CM is a complex disease with a probable poligenic background, more genetic variants are likely to contribute to the susceptibility of the disease, suggesting that a large number of patients and controls are needed to achieve sufficient power to detect a genetic association.

In recent years it seems increasingly clear that epigenetic processes play an important role in a wide variety of multifactorial diseases, including migraine. Although to date, there are not specific studies in CM patients, there is some evidence that neuronal activity occurring during cortical spreading depression, may cause epigenetic changes involved in neuronal plasticity, neuroprotection [59] and regulation of basal synaptic activity [60]. It is therefore conceivable that increased neuronal activity in patients with high frequency migraine may alter the cerebral epigenome, thereby promoting subsequent attacks of migraine and creating a cycle in which the epigenetic programming of genes and pathways underlying excitability are altered towards a more sensitive baseline [61]. Some of the SNPs associated with migraine involved genes related to epigenetic processes, as well as epigenetic regulation of the Calcitonin Gene-Related Peptide (CGRP) gene. This evidence have given importance to the role of epigenetic processes in the pathophysiology and chronicity of migraine [62, 63].

### Molecular research and biomarkers

Biomarkers are defined as physical signs or laboratory measurements associated with a biological process with a diagnostic or prognostic utility [64]. Molecular biomarker levels can be measured in body fluids. Thus, on the one hand diagnostic biomarkers signal a pathogenic process and are linked to disease risk and on the other hand severity and therapeutic biomarkers indicate a treatment response and may predict the efficacy of an intervention [65].

Even though several studies have been done to find biomarkers in migraine [66], currently, there are no accepted biological markers for the diagnosis of migraine. The well-known marker CGRP is abundant in the body and has a wide distribution throughout the central and peripheral nervous systems [67]. It is known that CGRP plays an important role in the pathophysiology of migraine [68]. CGRP is a neuropeptide widely expressed in trigeminovascular system as well as numerous central nervous system sites associated with pain processing and migraine symptoms [68]. Furthermore, It plays a key role in the development of peripheral sensitization and enhanced abnormal pain sensitivity through a central pronociceptive role [69]. Elevated interictal CGRP levels have been proposed as a possible diagnostic biomarker for CM [70, 71]. Moreover, not all studies show a consistent increase in interictal serum CGRP levels in CM patients compared to EM patients or healthy controls [72]. Nonetheless, it has been shown that serum CGRP levels are associated with the response to treatment with Onabotulinumtoxin type A [73], which leads to a controversial discussion of a potential valuable biomarker for predicting treatment efficacy. Although the instability and short-life of the peptide and the variable detection methods complicates reliable and feasible measurement [68]. Even though CGRP may also contribute to the development of peripheral and central sensitization [74, 75], further research is necessary to confirm the potential of CGRP as biomarker in CM [66].

A second neuropeptide that is proposed as a biomarker for CM is Vasoactive Intestinal Peptide (VIP). Just like CGRP, VIP is released in the trigeminovascular system. Interictal serum levels of VIP have been found to be significantly increased in CM patients compared to healthy controls [73, 76] and, even though VIP serum levels seemed to be elevated compared to EM patients, this was not significant [76]. Furthermore, serum levels of VIP have been correlated with cranial autonomic parasympathetic symptoms in patients with CM [77]. Responders to Onabotulinum toxin type A had significantly higher VIP levels than non-responders. However, these results showed poor specificity [73]. In contrast to CGRP and VIP, another neuropeptide the “Pituitary Adenylate Cyclase-activating Peptide (PACAP), that is also released in the trigeminovascular system, was not altered during the interictal phase in CM patients [78].

It is known that some adipokines (such as leptin and adiponectin), interleukin 6 (IL-6) and tumor necrosis factor alpha (TNF-α), can act as mediators of inflammatory processes linked to persistence and progression of migraine [79]. Inflammatory mediators may decrease the threshold for the onset of a migraine attack and may also contribute to central sensitization as in the case of other pro-inflammatory cytokines [80–82]. Moreover, increased serum leptin was detected in CM patients [83]. Leptin levels are correlated with body mass index and TNF-α and IL-6 [75, 81]. Furthermore, serum total adiponectin and high molecular weight adiponectin levels were higher in CM [84], and were also elevated in both EM and CM interictal periods [84, 85]. Further evidence for the importance of adipokines in CM stems from the fact that CM seems to occur with higher incidence in obese people, with the risk of EM to CM progression being three or five times greater than in normal weight subjects [86]. Levels of the proinflammatory cytokine TNF-α have been found to be increased in cerebrospinal fluid (CSF) in treatment-resistant CM patients [87], while levels of somatostatin and glial cell line-derived neurotrophic factor (GDNF) were decreased in the CSF of patients with CM [88].

Another possible biomarker for CM is glutamate. Glutamate levels in the CSF are higher in patients with CM compared to controls [89], and glutamate levels measured in saliva have been found to be significantly increased in CM patients compared to patients with EM [90]. Moreover, prophylactic treatment using topiramate, amitriptyline, flunarizine or propranolol reduced plasma glutamate levels along with a reduction in the number of headache days per month, with no differences among the types of prophylaxis [91]. Therefore, glutamate could serve as a potential biomarker for CM.

Some other studies for migraine biomarkers include serotonin, S100β, neurokinin A and substance P. However, most of these studies focus on EM and results seem to be inconsistent [92–96].

Migraine-specific biomarkers are needed not only for the improvement of therapeutic approaches, but also for the development of new and personalized treatments. Multiple potential biomarkers for CM have been investigated so far, but further controlled clinical trials are still needed to investigate both their diagnostic and therapeutic value.

### Neurophysiology

Neuronal activity in migraine has been widely characterized through electrophysiological studies, which assess the brain spontaneous activity and evaluate its response to different stimuli [97]. Between them, evoked potentials to different sensory modalities (in particular somatosensory and visual), transcranial magnetic stimulation and magnetoencephalography studies have shown the most relevant findings [98]. Notwithstanding, the pathophysiology of migraine still remains not fully understood. Data from different studies are often difficult to compare because of methodological differences, patient’s heterogeneity and different points of evaluation thought the cycle of the migraine attack.

Neurophysiological studies have investigated the cortical excitability state in migraine, so-called migraine cortical “dysexcitability” [99]. Different pathophysiological mechanisms might coexist in migraine, possibly being either expression of increased cortical responsivity or compensatory mechanisms seeking to stabilize the cortical excitability level [100].

Experimental data form EM patients have shown that electrophysiological features of the migraineur’s brain fluctuates in relation with the cyclical recurrence of the migraine attack. Habituation is defined as a decremental response to repeated stimulations. Electrophysiological techniques in EM revealed interictal deficient habituation of any kind of sensory responses (except for olfactory stimulation) attributed to abnormal thalamo-cortical interactions that normalizes during the migraine attack [101]. Studies with repetitive transcranial magnetic stimulation (rTMS) have also reported interictal paradoxical cortical responses in reaction to both depressing or enhancing rTMS stimulation that changes up to the bending point of an attack when cortical responsivity behaves differently [102].

Compared to EM, CM patients have lower pain thresholds as measured on quantitative thermal and mechanical sensory test [103]. Studies using blink reflex showed a remote effect of C fiber activation by capsaicin that suggests impaired diffuse noxious inhibitory control, that selectively inhibits action of nociceptive neurons located in the nucleus of the descending trigeminal tract by remote noxious stimuli, in CM but not in EM [104]. But one of the most reproducible underlying features in CM is an increased cortical excitability that has been demonstrated by different study methods. Magnetic visual evoked responses in CM patients demonstrate lower phosphene thresholds, decreased cortical inhibition [105, 106] and persistent ictal-like excitability pattern of the visual cortex between migraine attacks which may implicate central inhibitory dysfunction [107]. The response pattern of the visual cortex in patients with CM is similar to that found during a migraine attack in patients with EM, both normal with regard to habituation and abnormal regarding amplitude of the evoked response after a low number of stimuli [107]. But habituation deficit reappears in CM patients who remitted to EM, suggesting that visual cortical excitability reflect the clinical status of migraine [108]. Similarly, it has been showed that response pattern of the somatosensory cortex to repeated somatosensory evoked potentials in CM patients is similar to that found during a migraine attack in EM patients: both habituates normally but with an initial sensitization response. Sensory sensitization may be explained by connections between the thalamus and cortex intensified in CM compared to EM between attacks [109]. These data support the fact that thalamocortical dysfunction might be associated with a progressive extension of an acute electrophysiological alteration up to a basal modification of neuronal activity.

In CM patients, rTMS applied to the primary motor cortex showed inhibitory responses resembling that observed in EM patients with high attack frequency evaluated interictally, and in patients in the ictal state, what may also be an expression of reduced inhibitory homeostatic responses [100].

Differences between episodic and CM may not be principally confined to the number of headache days per month, but instead reflect a more profound pathophysiological distinction [110]. Taken together, neurophysiological data can be considered as robust evidence for the cycling functional brain alterations as a prominent features of migraine pathophysiology, but mechanisms underlying progression are still unknown and whether the diffuse excitability change of CM brain is the cause or the consequence of migraine chronification process is not elucidated yet [109].

### Animal models

CM is classified as a single entity, so, specific animal models that mimic CM features have been developed to test preventative medications and investigate pathophysiological mechanisms of migraine transformation. Currently, there are several methods to induce headpain in animals but, because of the complexity of migraine, there is no unique animal model that replicates all components of CM, and current models focus on reproducing single phenotypic or endophenotypic features. It is possible to model CM using repeated stimuli that activates trigeminal nociceptors representing the episodic nature of migraine attacks. This includes epidural application of an inflammatory soup and intravenous infusion of glyceryl trinitrate (GTN) [111]. Transgenic animal models of CSD induction had not been completely validated for CM study. As mentioned before, one of the main features of migraine chronification is the sensitization of the trigeminothalamic pathways. Allodynia is a common symptom of migraine that has been correlated with central and peripherical sensitization, increased migraine frequency ant thus, chronification [112]. Due to its clinical translation in humans, trigeminal mechanical sensitivity measurement in animals using von Frey hair stimulation in facial or paws is considered one of the best strategies to determine pain sensitization, although nociceptive-related behavioral changes can be used [111].

Current animal models to study CM includes a mouse model involving the repeated intraperitoneal administration of GTN resulting in acute hyperalgesia, and a chronic basal hyperalgesia reduced by topiramate, but not sumatriptan that persists after the cessation on GTN [113]. Another model is based on repeated application of inflammatory soup onto the dura mater that induces allodynia and increase of nociceptive-related behavior that reduces after zolmitriptan administration [114].

A GTN model has been used to identify genes and biological processes impacted by chronification compared to controls. Differential gene expression in trigeminal ganglion and nucleus accumbens in response to NTG treatment has been demonstrated, including genes linked to glutamatergic and dopaminergic synapses and rhythmic process among others that could be involved in CM pathophysiology [115].

CM animal models have shown increased CGRP gene expression in rodents pain processing areas such as trigeminal nucleus caudalis [116, 117]. GTN induced model showed that animal behavioral changes in pain perception correlated with an increased gene expression of CGRP in the medulla-pons region, cervical spinal cord and trigeminal ganglia [118], while it has not been demonstrated after acute GTN administration [119], supporting CGRP contribution in central sensitization.

The BBB permeability during migraine attacks has been widely discussed, and there is data that supports [120, 121] and contradicts BBB disruption [122, 123] in EM but little is known about BBB permeability in CM patients. One study used the inflammatory soup rat model of trigeminal allodynia, to determine the impact of repeated dural inflammatory stimulation on BBB permeability. and demonstrated a significant increase in BBB permeability and astrocyte and microglial activation in the trigeminal nucleus caudalis during the chronic phase after repeated infusion [124]. These findings could be in line with inflammatory pain states producing significant changes in the BBB permeability but need further confirmation [125].

In animals, chemical activation and sensitization of meningeal sensory neurons can lead to activation and sensitization of central trigeminal neurons that receive convergent input from the dura and skin [126]. Continuous stimulation of trigeminal neurons during repeated migraine attacks lead to changes in activity of intracellular signalling molecules that are relevant to pain and increase expression of inflammatory cytokines in the trigeminovascular system, thereby promoting the chronification process [127]. Using inflammatory models ﻿the findings indicate that inflammatory pathways and overexpression of CGRP in nociceptive neurons, could participate in the generation of pain hypersensitivity [128]. Transgenic mice sensitized to CGRP through elevated expression of a CGRP receptor could be used in the future to test the hypothesis of chronic CGRP-induced neurogenic neuroinflammation [129]. Furthermore, the central sensitization phenomenon underlines connectivity changes through synaptic plasticity. Actually, a rat model based on repeated stimulations with inflammatory soup has showed that central sensitization correlates to an increase of the synaptic efficiency through NR2B-pTyr expression. This protein has been already related to the regulation of the synaptic plasticity in the central sensitization in this CM rat model [130].

Preclinical research with animal models has provided valuable information about the mechanism of action on preventive treatments. Treatments that have proved efficacy in migraine patients, have been shown to prevent mechanical hyperalgesia in animal models [113, 131]. For example, botulinum toxin could act peripherally inhibiting the release of a variety of neurotransmitters which are known to be key signaling molecules in CM including CGRP [132, 133], so animal pre-treatment with botulinum toxin can prevent mechanical sensitization inhibiting mechanical nociception in peripheral trigeminovascular neurons [134]. For example, the mechanism of action of noninvasive vagus nerve stimulation for migraine treatment have also been investigated in the inflammatory soup model showing a decrease in periorbital sensitivity after de vagal stimulation [135].

In summary, only a few CM models are available today that can mimicmigraine features observed in accordance with clinical findings. However, as these animal models for long-term activation of the trigeminovascular system can only show unique phenotypical features of CM, like allodynia or photophobia so, it is important to stress that these are not a model of the migraine spectrum. Ideally, specific models should be able to show the broad spectrum of symptoms developed by migraine patients.

### Neuroimaging

Migraine is thought to conform a disease spectrum with symptoms gradually evolving from the episodic to chronic forms that is characterized by several neurophysiological changes. Increasingly more studies suggest that these changes may be evaluated using neuroimaging techniques, which try to understand central underlying pathophysiological mechanisms [136, 137]. Changes shown by these studies may reflect chronic pain susceptibility or be a consequence of recurrent migraine attacks [138, 139].

Structural differences measured on magnetic resonance imaging (MRI) have been found between migraine patients and normal healthy controls [140]. Some neuroimaging studies performed in EM patients have shown structural differences correlated with headache frequency, that could be understood as indirect markers of migraine chronification. Patients with a high frequency of migraine attacks have thicker somatosensory cortex, anterior cingulate cortex and the inferior temporal gyrus compared with patients with a low frequency of attacks [141]. The frequency of migraine attacks was also correlated with cortical thickness in the left middle frontal gyrus and in the left central sulcus [142].

Studies performed specifically in CM patients have shown volumetric changes in amygdala, putamen, hippocampus and brainstem areas [138, 143]. The volume of hippocampus and amygdala seems to change with headache frequency. The hippocampus is thought to be involved in a maladaptive stress response, while the amygdala plays a central role in emotions, fear conditioning, processing of prolonged nociceptive inputs, and development of sensitization. Compared to healthy controls grey matter volume of the amygdala and putamen is increased in CM patients [138]. Another study also shows an increasing in volume of the hippocampus and left amygdala that positively correlates with frequency followed by a decrease when the headache becomes chronic [144]. Patients with smaller hippocampus may have a higher vulnerability to stress, stress related disorders and persistent pain [144].

Taken together, these structural differences seem consistent enough that a model can be performed to accurately differentiate between chronic, episodic and healthy controls [145].

Structural differences have also been found in the periaqueductal gray (PAG) of CM patients. PAG is a structure that plays an important role in the modulation of nociceptive stimuli from the trigeminal nucleus and it is considered a key structure of migraine. The volume of periaqueductal gray matter is increased in EM patients in comparison to healthy controls but decreases again in CM patients [110]. It has also been demonstrated the presence of iron accumulation in the PAG as well as in the red nucleus in CM patients compared to EM patients. This accumulation can be due to recurrent attacks with secondary damage since biomarkers of endothelial dysfunction endothelial and blood brain barrier (BBB) molecular disruption are also elevated in this group. This could lead to progressive dysfunction and chronification, but this stays speculative since iron accumulation increases with age, while migraine decreases with age [146].

Another common structural finding in migraine patients are white matter lesions (WML) [147]. The presence of WML has been related to disease duration and the attack frequency [148] but there are no specific studies that evaluate the evolution of WML during transformation from episodic to CM. Studies using diffusion tensor imaging (DTI) did not find differences between chronic and EM patients due to microstructural white matter changes [149].

A promising way to explore the underlying anatomy and pathophysiology regarding the chronification of migraine is functional MRI. Functional MRI is an important tool to study both brain structure and brain function in one single technique [150]. Recent studies point to a key role for the brainstem and hippocampus in the first phase of a migraine attack [151]. The limbic system, on the other hand, seems to have an important role in pain networks in CM [140].

The amygdala (part of the limbic system) has a uniquely increased connectivity with several parts of the brain in patients with CM. This finding has not been replicated in patients with EM, suggesting an important limbic pain network dysfunction specifically in migraine but not seen in other chronic pain syndromes [152]. The hypothalamus shows stronger activation in the CM patient than in EM patients in response to painful trigeminal stimulation but also during a migraine attack [153]. The posterior part of the hypothalamus seems to be involved in the acute pain stage, while the anterior part seems to be involved in the attack generation and preictal phase and also migraine frequency, suggesting that it plays an important role in chronification [153]. This is supported by the fact that there is an increased connectivity between the anterior hypothalamus and spinal trigeminal nucleus in the CM compared to the episodic group [154]. A study that have compared EM and CM patients using resting state technique, points to stronger connectivity in the pain matrix of CM patients that might play a role in migraine chronification [155].

At this stage, there is still a far way to go until we find a neuroimaging marker for CM. Although, the results from neuroimaging studies in CM provide light to which structures or networks could be involved in the chronification process.

## Conclusions

CM patients show differences compared to EM patients and controls. EM patients with clinical factors associated to chronification may be on a higher risk for transformation, so it is important to screen for clinical risk factors as well as educate and treat modifiable factors in order to prevent transformation. Although studies using different approaches have demonstrated functional and structural differences between CM and EM patients, key structures and networks involved in the chronification phenomena, and the pathophysiology of migraine transformation are not fully understood. The changes shown may reflect migraine transformation susceptibility or be a consequence of recurrent migraine attacks. Taking this into account, the findings in this review do seem to point towards general changes in excitability of the central and peripheral nervous system. For example, increased levels of glutamate in the CSF, central sensitization, altered habituation to sensory stimuli, impaired cortical inhibition and furthermore when investigating magnetic visually evoked responses, and even predicting the state of chronification based on structural imaging, are all compatible with a hypothesis of central and peripheral altered excitability being pivotal changes happening in CM. Whether this would be part of the cause for, or a consequence of chronification remains to be elucidated.

## Data Availability

Not applicable.

## Abbreviations

AMPP: American Migraine Prevalence And Prevention
BBB: Blood Brain Barrier
CDH: Chronic Daily Headache
CGRP: Calcitonin Gene-Related Peptide
COMT: Catechol-O-Methyltransferase
CSF: Cerebrospinal Fluid
CM: Chronic Migraine
EM: Episodic Migraine
GTN: Glyceryl Trinitrate
GWAS: Genome-Wide Association Study
IBMS: International Burden Of Migraine Study
ICHD-3: International Classification Of Headache Disorders
IL-6: Interleukin 6
MRI: Magnetic Resonance Imaging
NSAIDs: Nonsteroidal Anti-Inflammatory Drugs
PACAP: Pituitary Adenylate Cyclase-Activating Peptide
rTMS: Repetitive Transcranial Magnetic Stimulation
SNPs: Single Nucleotide Polymorphisms
TNF-α: Tumor Necrosis Factor Alpha
VIP: Vasoactive Intestinal Peptide
